# P-1617. Longitudinal SARS-CoV-2 Surveillance in a University Cohort: Antigen Assay Performance, Pooling Efficiency, and Epidemiological Takeaways

**DOI:** 10.1093/ofid/ofaf695.1794

**Published:** 2026-01-11

**Authors:** Joseph W K Stuart, Gregory L Damhorst, Kaleb McLendon, Aneksi Ellis, Matthew J Denius, Essandoh F Abaidoo, John Roback

**Affiliations:** Emory University School of Medicine, Atlanta, GA; Emory University, Atlanta, GA; Emory University, Atlanta, GA; Emory University, Atlanta, GA; Emory University, Atlanta, GA; Emory University, Atlanta, GA; Emory University School of Medicine, Atlanta, GA

## Abstract

**Background:**

Global shortages of RT-PCR reagents during the COVID-19 pandemic necessitated the adoption of alternative diagnostic and screening approaches. We implemented a saliva antigen-based surveillance program at Emory University to manage the spread of infection and gather epidemiological data.

**Figure 1.**
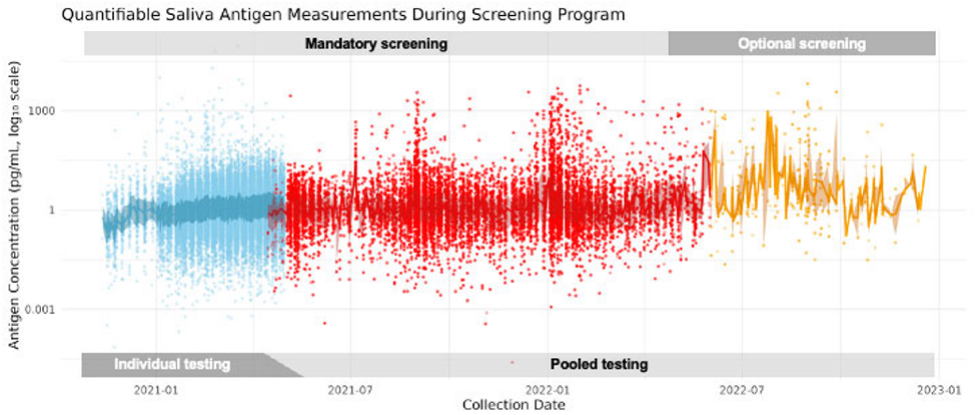
Quantifiable Saliva Antigen Measurements During Screening Period Weekly saliva samples from undergraduates (Nov 2020–Dec 2022) were assayed for SARS-CoV-2 nucleocapsid antigen (pg/mL, log_10_ scale). Points are colored by testing phase: individual (blue), pooled (red), and optional (orange). Solid lines trace the median antigen concentration in each phase, with shaded ribbons showing the interquartile range. Gray bands above and below the plot denote periods of mandatory individual screening, pooled screening, and optional surveillance.

**Figure 2.**
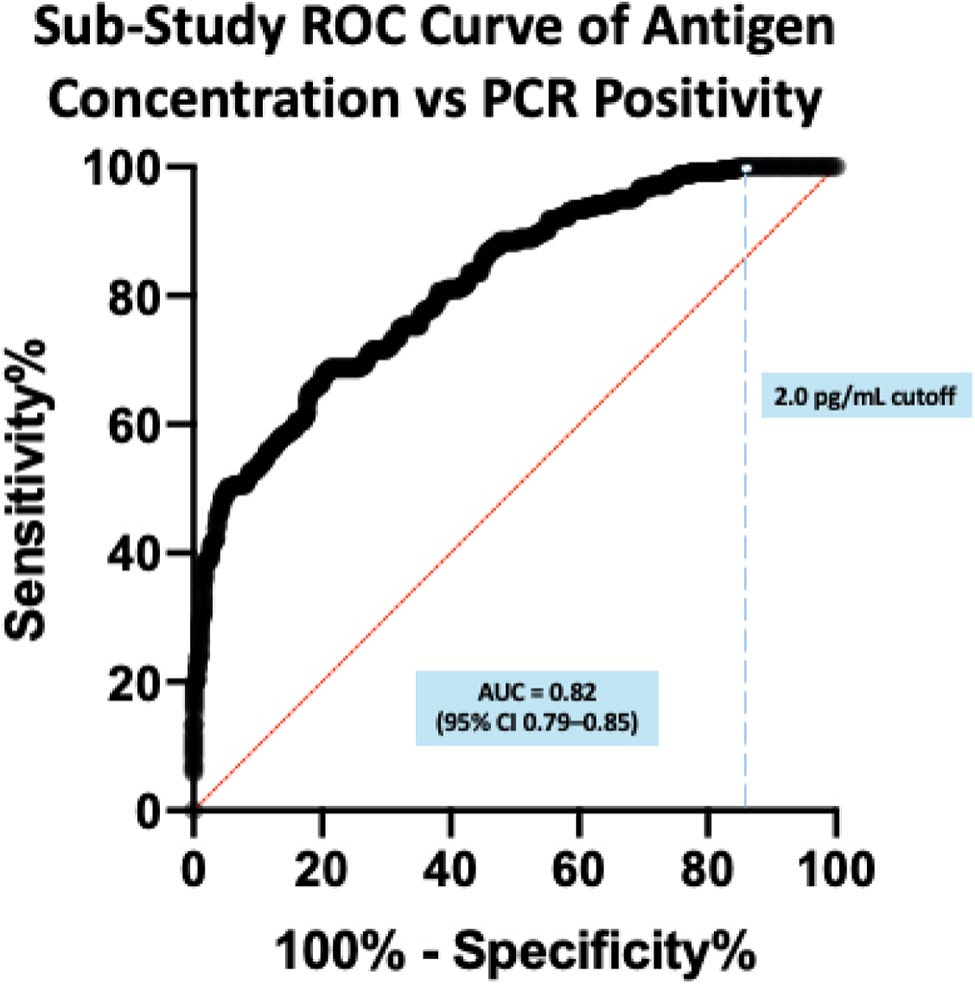
Sub-Study ROC Curve of Antigen Concentration vs. PCR Positivity ROC curve from the sub-study (N = 943) comparing Simoa antigen concentration to RT-PCR positivity. Sensitivity is plotted against 1 – specificity on a 0–100 % scale. Area under the curve = 0.82 (95 % CI 0.79–0.85). The dashed vertical line marks the 2.0 pg/mL cutoff, which yields 99.6 % sensitivity and 15.4 % specificity.

**Methods:**

From November 2020 through December 2023, participants submitted saliva samples as frequently as weekly, which were tested for SARS-CoV-2 nucleocapsid antigen by single molecule array assay (Simoa) on the Quanterix HD-X instrument. Specimens were tested individually from January 2021 – May 2021, and in pools of three specimens beginning in April 2021. Threshold for positivity of 3 pg/ml (individual specimens) was initially used, followed by 2 pg/ml (pooled specimens) after an optimization sub-study during which all participants were tested with both Simoa and RT-PCR and an ROC analysis was performed. Specimens or pools above the antigen threshold were reflexed to individual specimen RT-PCR for confirmation.

**Results:**

21,546 participants submitted 182,882 samples during the program period (Figure 1). 7,208 of 97,441 (7.4%) individual specimens were above antigen threshold, of which 298 (4.36%) tested positive by RT-PCR. 10,618 of 78,834 (13.47%) participants in three-specimen pools were above antigen threshold ( >2 pg/ml), of which 1,248 (11.6%) of individual presumptive positive participants tested positive by RT-PCR. During the optimization sub-study of 943 participants, 243 were positive by RT-PCR and 700 were negative. Area under the ROC curve was 0.82 (Figure 2). The revised threshold of 2 pg/mL for pooled samples corresponded to a sensitivity of 99.6% and specificity of 15.4%.

**Conclusion:**

We refined a saliva antigen screening program with three-specimen pooling and a 2 pg/ml threshold, which achieved >99 % sensitivity and efficient resource use, which identified SARS-CoV-2 cases (AUC 0.82 in our sub-study) fulfilling Emory University's surveillance needs while providing interesting epidemiological insights. This scalable framework supports rapid, high-throughput pandemic surveillance despite resource limitations.

**Disclosures:**

All Authors: No reported disclosures

